# Fulminant Thromboinflammatory Syndrome Following an Influenza-like Illness in an Adolescent: Clinical Insights from a Case Report

**DOI:** 10.3390/ijms27167456

**Published:** 2026-08-20

**Authors:** Mircea Stoian, Cristina Elena Garbovan, Sergio Rares Bandila Bandila, Attila Frigy, Adina Stoian, Dragoș-Florin Babă, Mircea Cătălin Coșarcă, Leonard Azamfirei

**Affiliations:** 1 Department of Anesthesiology and Intensive Care Medicine, George Emil Palade University of Medicine, Pharmacy, Science and Technology of Târgu Mureș, 540103 Târgu Mureș, Romania; mircea.stoian@umfst.ro (M.S.);; 2 Department of Infectious Diseases, George Emil Palade University of Medicine, Pharmacy, Science and Technology of Târgu Mureș, 540136 Târgu Mureș, Romania; 3 Orthopedic Surgery and Traumatology Service, Marina Baixa Hospital, Avinguda Alcade En Jaume Botella Mayor, 03570 Villajoyosa, Spain; sergiob1976@gmail.com; 4 Department of Internal Medicine IV, George Emil Palade University of Medicine, Pharmacy, Science and Technology of Târgu Mureș, 540136 Târgu Mureș, Romania; attila.frigy@umfst.ro; 5 Department of Pathophysiology, George Emil Palade University of Medicine, Pharmacy, Science and Technology of Târgu Mureș, 540136 Târgu Mureș, Romania; 6 Department of Cell and Molecular Biology, George Emil Palade University of Medicine, Pharmacy, Science and Technology of Târgu Mureș, 540142 Târgu Mureș, Romania; dragos-florin.baba@umfst.ro; 7 Department of Anatomy, George Emil Palade University of Medicine, Pharmacy, Science and Technology of Târgu Mureș, 540139 Târgu Mureș, Romania; catalin.cosarca@umfst.ro; 8 Clinic of Vascular Surgery, Mureș County Emergency Clinical Hospital, 540136 Târgu Mureș, Romania

**Keywords:** thromboinflammation, disseminated intravascular coagulation, anti-platelet factor 4/heparin antibodies, endothelial activation, influenza-like illness, Protein C pathway

## Abstract

Infection-associated thromboinflammation may result from dysregulated interactions between innate immune activation, endothelial activation, and coagulation. Although influenza-like illnesses are generally self-limited in young individuals, severe respiratory infections have been associated with thrombo-inflammatory complications involving coagulation dysregulation and endothelial activation. We describe an 18-year-old previously healthy woman who developed extensive left iliofemoral deep vein thrombosis, bilateral pulmonary embolism, and progressive systemic venous and intracardiac thrombosis following an influenza-like illness. Despite therapeutic anticoagulation, the clinical course was complicated by acute compartment syndrome, progressive tissue ischemia, circulatory failure requiring vasopressor support, multiple organ dysfunction, and ultimately below-knee amputation. Laboratory investigations revealed thrombocytopenia, severe hypofibrinogenemia, markedly elevated D-dimer levels, reduced Protein C activity, and increased von Willebrand factor antigen (>500%), consistent with overt disseminated intravascular coagulation and endothelial activation. A positive anti-PF4/heparin immunoassay raised concern for heparin-induced thrombocytopenia; however, the subsequent clinical and laboratory evolution supported overt DIC as the predominant consumptive coagulopathy, while HIT could neither be confirmed nor definitively excluded. This case highlights a fulminant thrombo-inflammatory syndrome temporally preceded by an influenza-like illness of unconfirmed etiology and the diagnostic challenges posed by overlapping features of thrombotic-predominant overt disseminated intravascular coagulation and anti-PF4/heparin antibody positivity.

## 1. Introduction

Acute viral respiratory infections are generally self-limited in young individuals. However, severe viral infections may induce systemic complications involving endothelial activation, coagulation abnormalities, thromboembolic events, and multiple organ failure [1,2]. Increasing evidence suggests that viral infections may trigger a dysregulated interaction between innate immunity, endothelial activation, and coagulation, resulting in a thrombo-inflammatory state associated with significant morbidity and mortality [1,3].

The concept of immunothrombosis has emerged as a key mechanism linking innate immune activation to intravascular thrombosis. Current evidence indicates that immunothrombosis represents a coordinated multicellular host-defense response involving endothelial cells, platelets, neutrophils, monocytes, complement activation, coagulation proteases, and inflammatory mediators. Under physiological conditions, these tightly regulated interactions contribute to pathogen containment while preserving vascular integrity. However, excessive or dysregulated activation transforms this protective response into pathological thromboinflammation, promoting intravascular thrombin generation, platelet activation, fibrin deposition, microvascular dysfunction, and ultimately organ failure [1,4,5,6,7].

Endothelial activation plays a central role in this process through glycocalyx degradation, increased vascular permeability, leukocyte adhesion, and massive release of von Willebrand factor (vWF) [1,5,8]. This process reflects systemic endotheliopathy, characterized by Weibel–Palade body exocytosis, loss of endogenous anticoagulant activity, dysregulated endothelial–leukocyte interactions, and sustained endothelial activation. Increasing evidence suggests that endotheliopathy is not merely a consequence of severe inflammation but a central driver of thrombo-inflammatory disease progression [9]. Recent evidence further emphasizes that immunothrombosis should be viewed as an integrated cellular network involving endothelial cells, neutrophils, monocytes, platelets, complement activation, and coagulation pathways rather than as an isolated coagulation abnormality [1].

In critically ill patients, dysregulated immunothrombosis may progress to DIC, particularly in severe infection-associated thrombo-inflammatory states. Sepsis-induced coagulopathy represents one recognized pathway leading to overt DIC [5,8,10]. The updated ISTH Scientific and Standardization Committee framework recognizes DIC as a dynamic intravascular process characterized by systemic coagulation activation, dysregulated fibrinolysis, endothelial activation, and progressive organ dysfunction. It distinguishes early-phase from overt DIC and recognizes thrombotic and hemorrhagic phenotypes according to the predominant pathophysiological mechanisms [11]. Among the endogenous anticoagulant systems, the Protein C pathway is an important regulator of endothelial homeostasis and thrombo-inflammatory responses [5,12].

One of the hallmarks of sepsis-induced coagulopathy and overt disseminated intravascular coagulation is disruption of the Protein C anticoagulant axis and other physiological anticoagulant pathways. Reduced Protein C activation contributes to excessive thrombin generation, endothelial activation, and progressive organ dysfunction [5,12]. Beyond its anticoagulant function, activated Protein C exerts anti-inflammatory, cytoprotective, and endothelial barrier-stabilizing effects through endothelial protein C receptor (EPCR)-dependent signaling, thereby limiting leukocyte adhesion, vascular permeability, and thrombo-inflammatory amplification [1,12].

In parallel, excessive release of von Willebrand factor and dysregulation of the von Willebrand factor–ADAMTS13 axis have been increasingly recognized as markers of endothelial injury and thrombo-inflammatory disease severity. Together, impaired Protein C pathway activity and endothelial activation are thought to represent key mechanisms linking inflammation, coagulation dysregulation, and organ dysfunction in severe thrombo-inflammatory states [13,14,15].

A scoping review identified 58 reported cases of thrombosis in patients with laboratory-confirmed influenza, including 21 pulmonary emboli, 12 deep-vein thromboses, and only 3 cases combining both conditions [16]. We found no report reproducing the complete constellation of multisite thrombosis, limb-threatening venous ischemia, subsequent overt DIC, and anti-PF4/heparin antibody positivity observed in our patient. Nevertheless, because the preceding influenza-like illness was not microbiologically confirmed, an influenza-associated etiology cannot be established. Differentiation between overt DIC and HIT may also be challenging in critically ill patients presenting with thrombosis, thrombocytopenia, and a positive anti-PF4/heparin immunoassay [5,17,18].

We report the case of an 18-year-old previously healthy woman who developed a fulminant thrombo-inflammatory syndrome following an influenza-like illness, characterized by extensive venous thromboembolism, progressive multisite thrombosis, compartment syndrome, thrombotic-predominant overt disseminated intravascular coagulation, circulatory shock requiring vasopressor support, multiple organ dysfunction, and ultimately below-knee amputation. To place this case in clinical context, we provide a focused narrative review of the literature addressing the pathophysiological mechanisms and diagnostic challenges associated with severe infection-associated thrombo-inflammatory syndromes.

## 2. Focused Literature Search for Mechanistic and Diagnostic Context

A focused PubMed/MEDLINE search was conducted from database inception to 31 July 2026 to identify publications relevant to the mechanistic interpretation and case-based differential diagnosis of the present case. The search combined the terms “influenza,” “influenza-like illness,” and “respiratory viral infection” with “thrombosis,” “venous thromboembolism,” “immunothrombosis,” “endothelial activation,” “disseminated intravascular coagulation,” “sepsis-induced coagulopathy,” “Protein C,” “von Willebrand factor,” “anti-PF4 antibodies,” and “heparin-induced thrombocytopenia.”

English-language original studies, case reports, case series, relevant mechanistic studies, reviews, clinical guidelines, and consensus statements were considered when they directly addressed the clinical or mechanistic features relevant to the case. Titles and abstracts were assessed for relevance, followed by full-text evaluation when appropriate. Reference lists of selected publications were also screened.

## 3. Case Presentation

### 3.1. Patient Information

An 18-year-old previously healthy woman was admitted to Mureș County Clinical Hospital following several days of fever, myalgia, fatigue, and progressive respiratory symptoms consistent with an influenza-like illness. Her condition subsequently deteriorated, with the development of dyspnea and increasing pain and swelling of the left lower extremity, prompting emergency hospitalization. She had no history of venous thromboembolism, autoimmune disease, malignancy, chronic inflammatory disorders, recent surgery, hormonal therapy, or known inherited thrombophilia. She was a non-smoker, had no previous exposure to heparin, and pregnancy was excluded at presentation. No relevant family history of thrombotic disease was reported.

### 3.2. Initial Presentation and Diagnostic Evaluation

At admission, the patient presented with persistent fever (38.4 °C), progressive dyspnea, and increasing pain and swelling of the left lower extremity following a recent influenza-like illness. On physical examination, she appeared acutely ill, with tachypnea, peripheral cyanosis, cold sweating, an oxygen saturation of 90% on room air, and a body mass index of 21.1 kg/m^2^. The combination of respiratory symptoms and acute unilateral lower-limb swelling prompted immediate evaluation for venous thromboembolism.

On 19 January 2026, a multiplex real-time RT-PCR assay was performed using the Sansure SARS-CoV-2, Influenza Virus and Respiratory Syncytial Virus Multiple Nucleic Acid Diagnostic Kit on a QuantStudio 5 Real-Time PCR platform. SARS-CoV-2, influenza A/B, and respiratory syncytial virus were negative. The exact symptom-onset date and respiratory specimen type could not be reliably recovered from the available records. Testing was performed eight days after the first documented medical evaluation on 11 January and on hospital day 5 after admission on 15 January. No respiratory viral serology or extended respiratory pathogen panel was documented. Consequently, the causative agent could not be retrospectively identified, and the preceding syndrome was classified clinically as an influenza-like illness of unconfirmed etiology.

Initial laboratory investigations demonstrated significant coagulation abnormalities, prompting an urgent multidisciplinary diagnostic work-up. Contrast-enhanced imaging studies of the thorax and peripheral vascular system were therefore performed to establish the extent of the disease and guide further management.

### 3.3. Imaging Findings

CT angiography on 19 January 2026 demonstrated complete occlusion of the left pulmonary artery, additional right segmental pulmonary emboli, and left common iliac vein thrombosis. Bilateral ground-glass opacities and consolidations were compatible with pulmonary infarction and/or inflammatory changes.

Follow-up CT angiography on 4 February demonstrated persistent pulmonary arterial thrombosis with pulmonary infarctions and thrombotic extension involving the superior vena cava, right atrium, infrarenal inferior vena cava, left renal vein, left iliac veins, and left femoral venous axis. Diffuse left-thigh soft-tissue edema was also present. On 13 February, CT demonstrated persistent inferior vena cava and left iliac thrombosis, extensive limb edema, and multiple rim-enhancing intramuscular collections concerning for secondary infection, with preservation of the arterial axis. Right internal jugular vein thrombosis, documented on 19 February after central venous catheter placement, was considered potentially catheter-associated.

**Figure 1:**
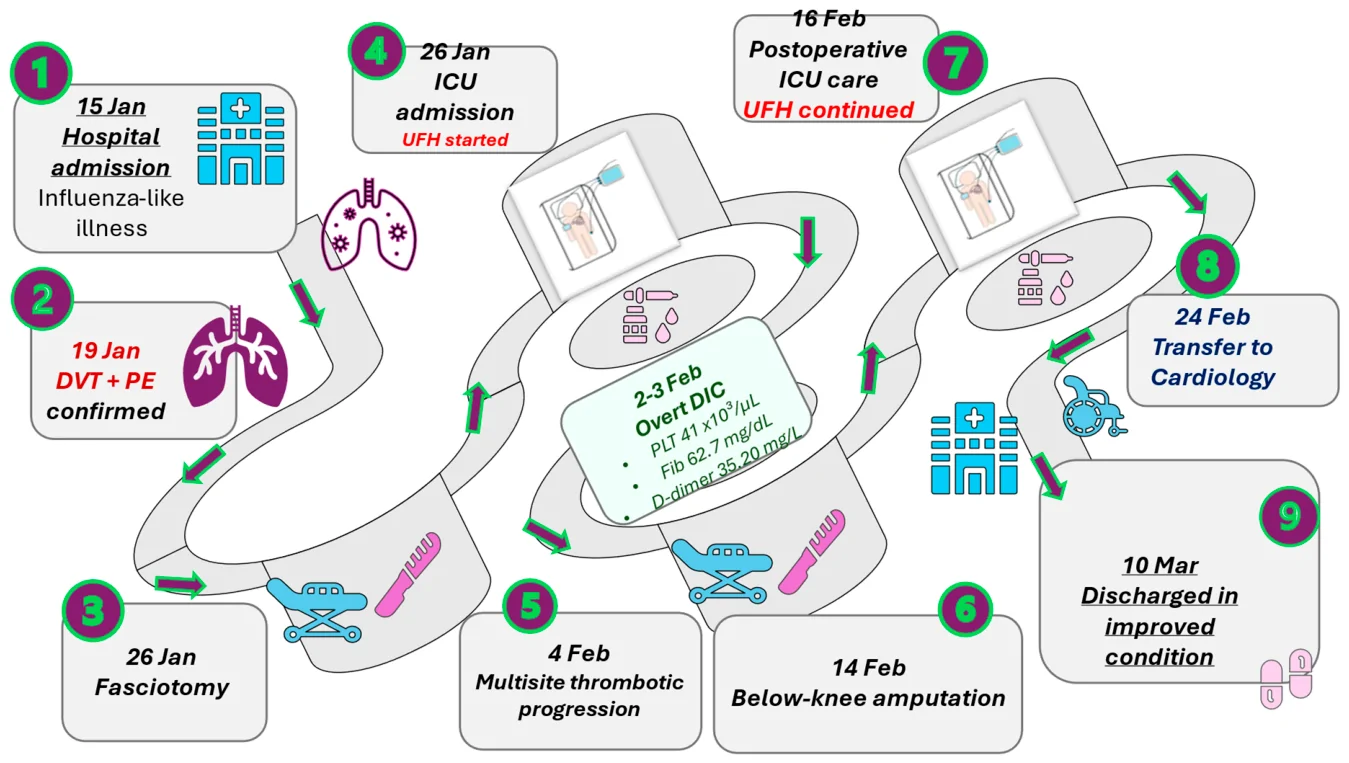
Clinical timeline of major diagnostic findings, therapeutic interventions, and clinical outcomes during hospitalization. Abbreviations: DIC—disseminated intravascular coagulation; DVT—deep vein thrombosis; ICU—intensive care unit; PE—pulmonary embolism; PLT—platelet count; UFH—unfractionated heparin. Notes: LMWH was initiated on 19 January, with the final dose administered on 26 January at 06:00. The anti-PF4/heparin sample was collected on 26 January at 12:30, and the positive result was reported on 5 February. Continuous intravenous UFH was initiated on 26 January at 21:00 and continued until 24 February, followed by apixaban. Overt DIC was documented on 3 February, with an ISTH score of 7. Numbers 1–9 indicate the chronological sequence of the major clinical events; green arrows indicate the direction of temporal progression; pictograms illustrate the corresponding clinical events or interventions; and red text highlights key thrombotic findings and anticoagulation-related information. Additional details are provided in Supplementary Table S1.

**Figure 2:**
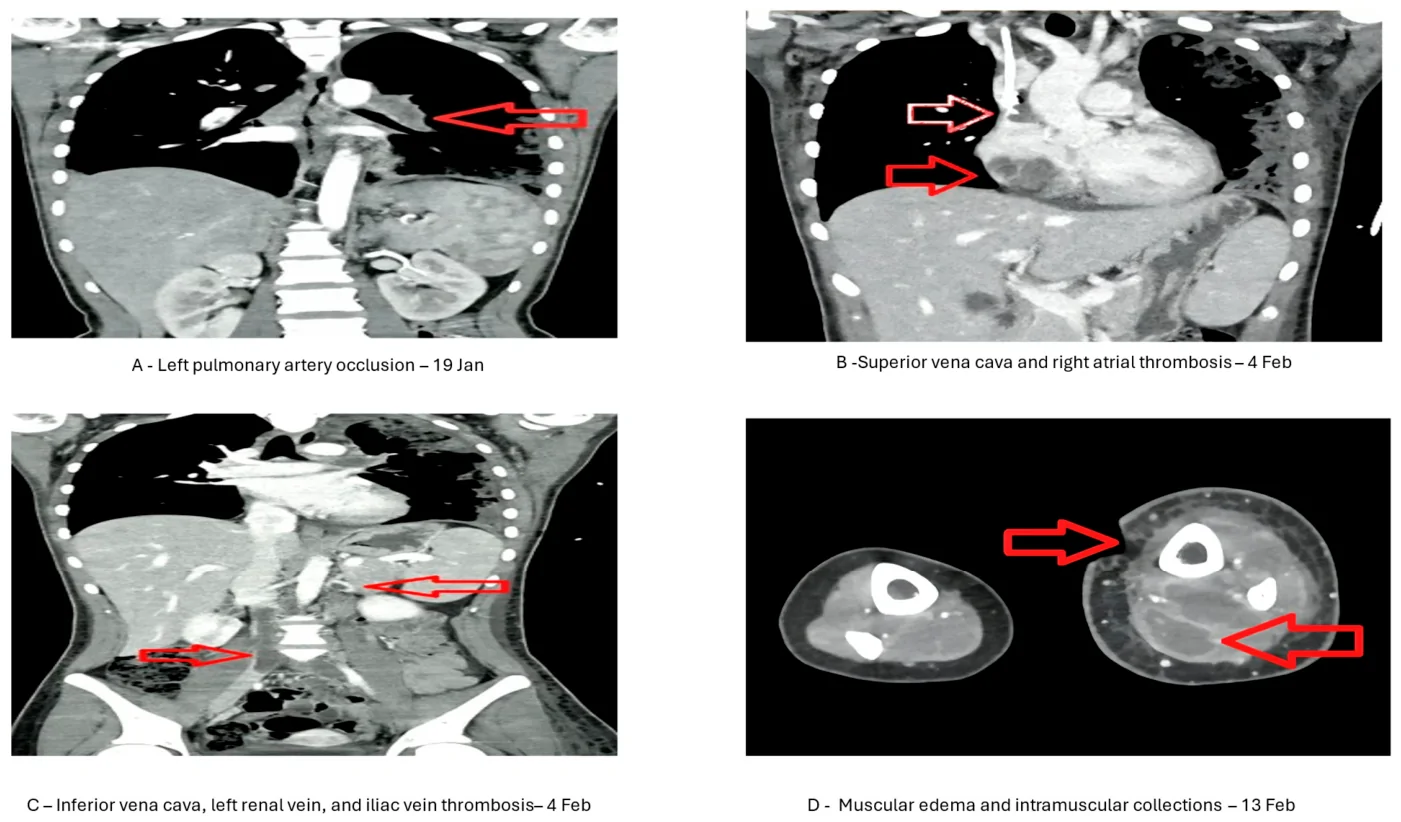
Serial contrast-enhanced CT images demonstrate progressive multisite thrombo-inflammatory disease despite therapeutic anticoagulation. (**A**) CT pulmonary angiography performed on 19 January 2026 showing complete occlusion of the left pulmonary artery with associated pulmonary infarction. (**B**) Coronal contrast-enhanced CT performed on 4 February 2026 showing thrombi involving the superior vena cava and right atrium. (**C**) Coronal contrast-enhanced CT performed on 4 February 2026 showing thrombosis of the inferior vena cava, left renal vein, and iliac vein. (**D**) Axial contrast-enhanced CT performed on 13 February 2026 showing diffuse muscular edema and multiple rim-enhancing intramuscular collections concerning for secondary infection. The arterial axis remained patent. Red arrows indicate the relevant radiological findings.

The principal clinical events are summarized in Figure 1, whereas the serial imaging findings are presented in Figure 2. Additional details are provided in Supplementary Table S1.

### 3.4. Clinical Course

**Figure 3:**
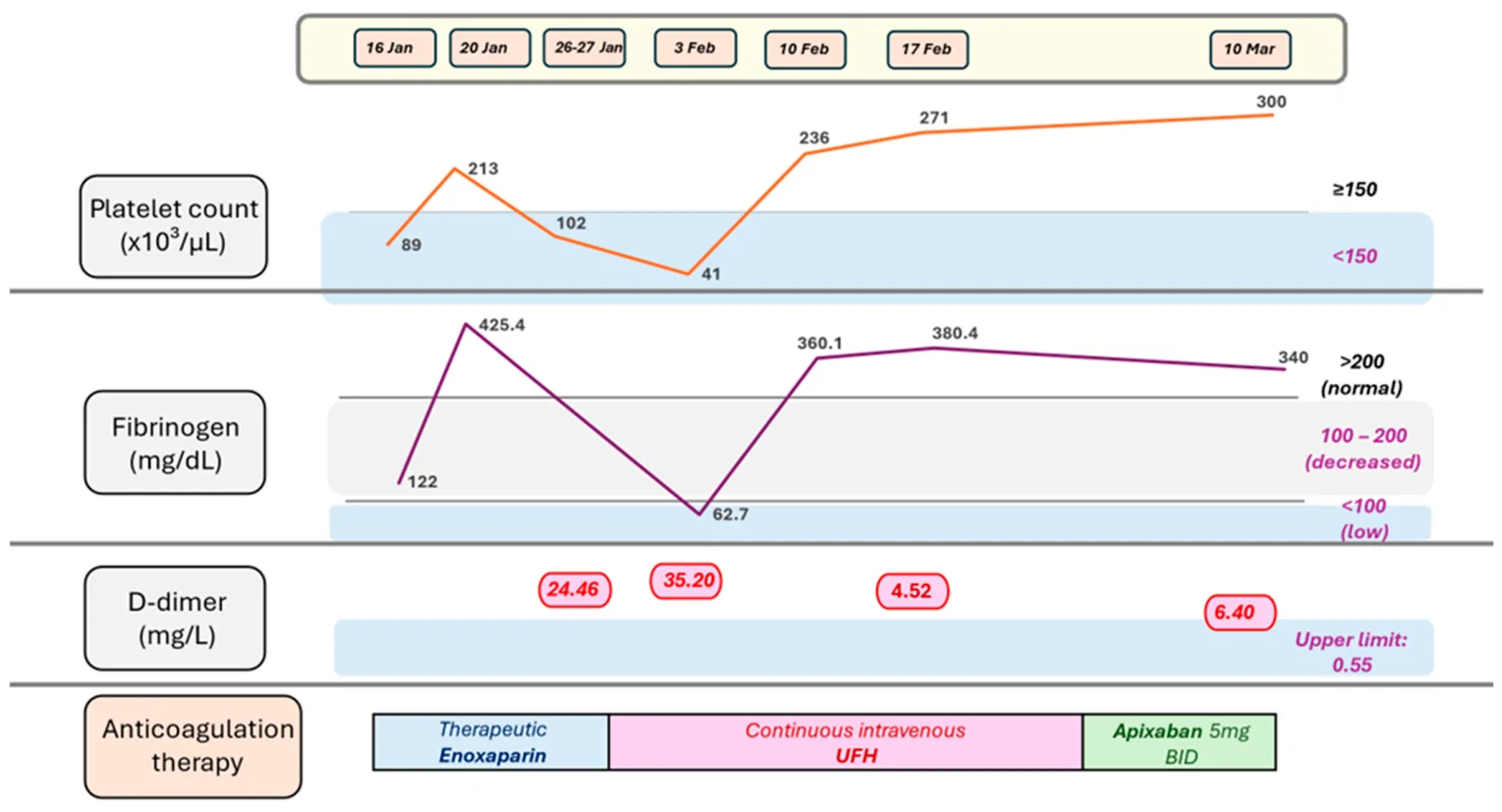
Temporal evolution of coagulation parameters, anticoagulant therapy, and major clinical events during hospitalization. Abbreviations: BID, twice daily; DIC, disseminated intravascular coagulation; PLT, platelet count; UFH, unfractionated heparin. Notes: LMWH was administered from 19 to 25 January, continuous intravenous UFH from 26 January to 24 February, and apixaban thereafter. The DIC panel corresponds to 3 February, when the ISTH overt DIC score was 7. Numbers indicate the measured laboratory values at the corresponding time points. Shaded areas and symbols indicate the reference thresholds. Red-outlined values indicate D-dimer concentrations above the upper reference limit, while the colored bars indicate the periods of anticoagulant therapy. Complete serial laboratory findings are presented in Table 1.

The hospital course was characterized by progressive thrombotic disease during anticoagulation. The evolution of coagulation parameters, anticoagulant therapy, and major clinical events is illustrated in Figure 3, with complete serial laboratory findings presented in Table 1.

Therapeutic-dose enoxaparin was administered from 19 January, with the final dose given on 26 January at 06:00. Continuous intravenous UFH was initiated on 26 January at 21:00 and continued until 24 February. Extensive iliofemoral thrombosis produced severe venous congestion, limb edema, and compartment syndrome. The initially preserved arterial axis supported critical venous outflow obstruction, rather than primary arterial occlusion, as the principal mechanism of limb ischemia.

Because of the rapid progression of limb ischemia, emergency fasciotomy was performed in an attempt to decompress the affected compartments and preserve limb viability. Despite surgical intervention, therapeutic anticoagulation, and intensive supportive care, tissue necrosis progressed, resulting in gangrene and irreversible loss of limb viability. The patient was therefore transferred to the Department of Vascular Surgery, where a below-knee amputation was performed, followed by transfer to the intensive care unit for continued multidisciplinary management.

During the ICU stay, the patient required invasive and subsequently noninvasive mechanical ventilation, vasopressor support, continuous venovenous hemodiafiltration, broad-spectrum antimicrobial therapy, transfusion support, metabolic and electrolyte correction, nutritional support, and serial clinical, laboratory, and imaging reassessment. The clinical course was complicated by circulatory shock, multiple organ dysfunction, extensive tissue ischemia, and progressive necrosis of the left lower limb.

Early coagulation abnormalities on 16 January did not establish overt DIC and temporarily improved by 20 January. From 31 January, thrombocytopenia and hypofibrinogenemia progressively worsened. On 2–3 February, platelets reached 41 × 10^9^/L, fibrinogen 62.2–62.7 mg/dL, D-dimer 35.20 mg/L, and PT 17.1 s.

Secondary bacterial infection was considered during the subsequent course. Procalcitonin exceeded 10 ng/mL on 6 February. Serial blood and urine cultures were negative, the sputum sample was non-diagnostic, and superficial purulent-secretion cultures obtained during antimicrobial therapy identified no specific pathogen. No deep-tissue, surgical, intramuscular collection, or amputation specimen cultures were available. CT on 13 February demonstrated extensive muscular injury and multiple rim-enhancing intramuscular collections concerning for secondary infection. Because these findings followed the initial thrombosis and overt DIC, bacterial infection may have contributed to later deterioration but was not established as the initial trigger.

The patient’s clinical condition gradually improved following multidisciplinary treatment. She was discharged on 10 March 2026 receiving therapeutic anticoagulation with apixaban 5 mg twice daily. Follow-up evaluations on 31 March, 30 April, and 17 June 2026 showed no evidence of recurrent thrombotic events. At the most recent follow-up, the platelet count had normalized (234 × 10^9^/L), the D-dimer level had decreased to 0.19 mg/L, and the patient had successfully completed rehabilitation with a below-knee prosthesis, regained independent ambulation, and resumed her usual daily activities. She remains on long-term anticoagulant therapy with apixaban 5 mg twice daily.

### 3.5. Serial Laboratory Findings During Hospitalization

Serial laboratory investigations were performed throughout hospitalization to monitor disease progression, coagulation abnormalities, inflammatory activity, organ dysfunction, and response to treatment. The temporal evolution of platelet count, fibrinogen concentration, D-dimer levels, anticoagulant therapy, and major clinical events are illustrated in Figure 3. The complete serial laboratory results, including hematological, coagulation, inflammatory, biochemical, and organ function parameters, are summarized in Table 1 according to the major phases of hospitalization.

### 3.6. Timing and Interpretation of Specialized Hemostatic Testing

The extended coagulation and thrombophilia panel was collected on 26 January 2026 at 12:30, after the final enoxaparin dose at 06:00 and before continuous intravenous UFH was initiated at 21:00. The last platelet count before heparin exposure was 163 × 10^9^/L on 18 January. Platelets peaked at 213 × 10^9^/L on 20 January and decreased to 136 × 10^9^/L on 23 January and 131 × 10^9^/L on 26 January. Initial DVT and pulmonary embolism preceded anticoagulation, whereas subsequent clinical thrombotic progression was documented by 25 January. The 4Ts score at sampling was 4: thrombocytopenia, 1 point; timing, 2 points; thrombotic progression, 1 point; and definite alternative causes, 0 points, indicating intermediate pretest probability.

The diagnostic work-up included assessment of natural anticoagulant pathways, coagulation factors, von Willebrand factor, antiphospholipid antibodies, anti-PF4/heparin antibodies, and selected inherited thrombophilia-associated genetic variants. The results are summarized in Table 2.

The presence of substantial heparin activity in the 26 January sample was supported by anti-factor Xa activity > 1.50 IU/mL and thrombin time > 160 s. Therefore, thrombin time, APCR, factor XI activity, and other potentially heparin-sensitive coagulation assays were interpreted with caution.

Serum obtained on the 26 January collection episode was stored frozen and referred through Bioclinica to a German reference laboratory for anti-PF4/heparin antibody testing. The reference laboratory registered the sample on 5 February at 11:02 and issued a qualitative positive result on the same day. The report identified antibodies against PF4/heparin complexes and recommended confirmation by HIPA when the clinical findings were atypical. The assay manufacturer, analytical platform, antibody class, quantitative signal strength, and cut-off value were not reported, and no functional platelet-activation assay was performed.

## 4. Discussion

To contextualize the present case, the relevant literature addressing immunothrombosis, endothelial activation, Protein C pathway impairment, disseminated intravascular coagulation, and anti-PF4/heparin antibody positivity was reviewed and integrated with the clinical findings. The present case illustrates a fulminant thrombo-inflammatory syndrome developing after an influenza-like illness in a previously healthy adolescent. The combination of extensive venous and intracardiac thrombosis, laboratory findings consistent with endothelial activation, disseminated intravascular coagulation, anti-PF4/heparin antibody positivity, and severe tissue ischemia culminating in limb amputation suggests the convergence of multiple interrelated pathogenic pathways. Understanding the interplay among these mechanisms may help explain the clinical evolution and diagnostic complexity observed in this patient.

### 4.1. Influenza-like Illness and the Subsequent Thrombotic Presentation

The clinical history began with an influenza-like illness of unconfirmed etiology. The exact symptom-onset date could not be reliably recovered. Multiplex RT-PCR performed on 19 January, eight days after the first documented medical evaluation, was negative for SARS-CoV-2, influenza A/B, and respiratory syncytial virus. Although delayed testing may have reduced diagnostic sensitivity, the negative result does not permit retrospective identification of the causative agent. The preceding illness was therefore retained as a clinical and temporal description rather than a confirmed etiological diagnosis [19,20,21].

**Figure 4:**
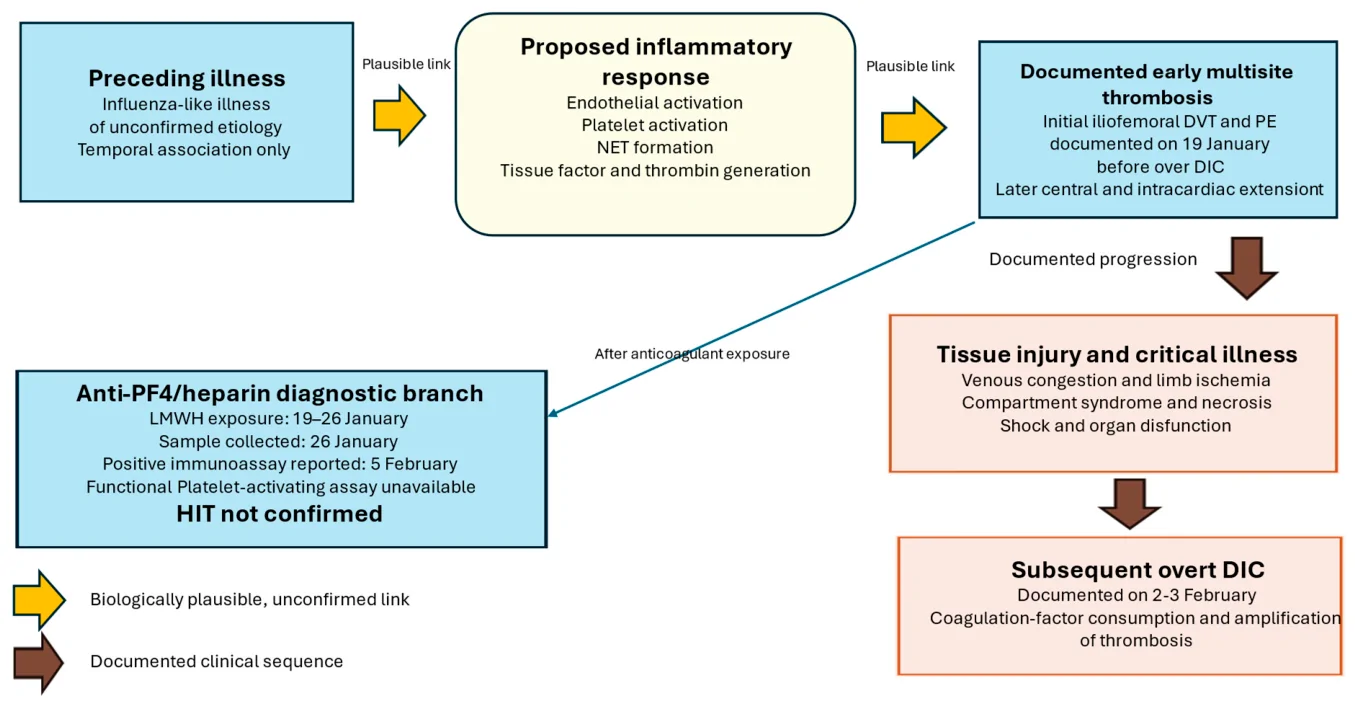
Case-based model of thrombo-inflammatory progression and anti-PF4/heparin antibody interpretation. Notes: Yellow arrows indicate biologically plausible but unconfirmed links, whereas brown arrows indicate the documented clinical sequence. The preceding influenza-like illness was of unconfirmed etiology and represents a temporal association. Initial multisite thrombosis preceded overt DIC, which was documented on 2–3 February 2026 and may subsequently have amplified thrombosis and coagulation-factor consumption. Anti-PF4/heparin immunoassay positivity did not demonstrate pathogenic platelet activation, and HIT was not confirmed because functional testing was unavailable. Abbreviations: DIC, disseminated intravascular coagulation; DVT, deep-vein thrombosis; HIT, heparin-induced thrombocytopenia; NETs, neutrophil extracellular traps; PE, pulmonary embolism; PF4, platelet factor 4.

Available evidence supports the biological plausibility of the individual components of this presentation, rather than the existence of a single established syndrome identical to the present case. In a scoping review, Rubino et al. identified 58 patients with laboratory-confirmed influenza and thromboembolic complications, including 21 with pulmonary embolism, 12 with deep-vein thrombosis, and three with both conditions [16]. In a population-wide cohort of 2.6 million individuals, Keene et al. found that hospitalization for pneumonia or influenza was followed by a markedly increased risk of venous thrombosis, particularly during the first four weeks [2]. Mechanistically, severe systemic inflammation may promote endothelial and platelet activation, platelet–leukocyte interactions, neutrophil extracellular trap formation, tissue-factor expression, and thrombin generation, thereby facilitating immunothrombosis [3]. Anti-PF4/heparin antibody positivity requires separate interpretation: Gendron et al. showed that antibodies detected in inflammatory and COVID-19 cohorts did not necessarily induce platelet activation [22], while current HIT guidance emphasizes integration of clinical probability, immunoassay results, and, when available, functional testing [17]. Accordingly, these studies support the plausibility of the individual thrombo-inflammatory findings but do not establish the preceding influenza-like illness as their cause or confirm clinically significant HIT in this patient (Figure 4).

Respiratory viral infections, including influenza, are increasingly recognized as a systemic disease capable of inducing profound vascular and hemostatic disturbances. Epidemiological studies have demonstrated a transient increase in the risk of venous thromboembolism, myocardial infarction, and ischemic stroke following influenza infection, supporting the concept that influenza should be regarded as a systemic thrombo-inflammatory disease rather than an isolated respiratory infection. Although venous thromboembolic events remain uncommon, accumulating evidence indicates that respiratory viral infections may act as transient triggers of thrombosis, particularly in susceptible individuals or in the presence of an exaggerated host inflammatory response [16]. These findings support the potential for severe respiratory infections to act as transient thrombotic risk factors but do not establish a causal relationship in the present patient.

In patients with laboratory-confirmed influenza, the proposed mechanisms underlying thrombosis include a close interaction between inflammation and coagulation. Viral infection promotes endothelial activation, increased tissue factor expression, platelet activation, cytokine release, and activation of the complement system. One of the earliest consequences of endothelial activation is Weibel–Palade body exocytosis, leading to the release of ultra-large von Willebrand factor multimers, increased leukocyte recruitment, enhanced platelet adhesion, and amplification of thrombin generation [1,3].

In parallel, neutrophil extracellular traps (NETs) amplify thrombin generation and fibrin deposition, a process currently referred to as immunothrombosis. Beyond their antimicrobial role, NETs provide a structural scaffold for platelet adhesion, activation of coagulation factors, and fibrin accumulation. Excessive NET formation has been implicated in infection-associated thrombosis, endothelial injury, and organ dysfunction, further reinforcing the concept of immunothrombosis as a dysregulated host response rather than a purely hemostatic abnormality [23,24,25,26,27]. Under physiological conditions these mechanisms contribute to host defense, but excessive activation may result in widespread thrombus formation and microvascular injury.

The evolution observed in our patient is consistent with dysregulated thromboinflammation. The rapid progression from an influenza-like illness to pulmonary embolism, extensive venous thrombosis, and subsequent intracardiac thrombosis, followed by severe coagulation abnormalities and limb-threatening ischemia, suggests that uncontrolled coagulation and endothelial activation may have contributed to disease progression. The markedly reduced Protein C activity together with markedly elevated von Willebrand factor antigen and activity are consistent with this hypothesis, although these abnormalities are not specific and may also reflect consumption and an acute-phase response [27,28,29].

### 4.2. Endothelial Activation and Dysregulation of the Protein C Pathway

Endothelial activation is increasingly recognized as a key mechanism linking inflammation and thrombosis in severe viral infections and sepsis. Following endothelial activation, the vascular surface shifts from an anticoagulant to a procoagulant phenotype, promoting platelet adhesion, thrombin generation, and microvascular thrombosis [8,29].

The Protein C pathway is one of the principal endogenous anticoagulant systems regulating this process. Reduced Protein C activity enhances thrombin generation and has been associated with disseminated intravascular coagulation, multiple organ dysfunction, and poor clinical outcomes. Likewise, markedly elevated von Willebrand factor (vWF) reflects endothelial activation and has emerged as a biomarker of thromboinflammation and disease [4,30,31]. The concomitant presence of markedly reduced Protein C activity and markedly elevated vWF suggests disruption of endothelial homeostasis, with sustained endothelial activation and impaired endogenous anticoagulant function. Rather than representing isolated laboratory abnormalities, these findings are consistent with a thrombo-inflammatory state involving endothelial activation and impaired endogenous anticoagulant regulation [1,12].

Beyond its anticoagulant function, activated Protein C exerts anti-inflammatory, cytoprotective, and endothelial-stabilizing effects through interactions with thrombomodulin and the endothelial protein C receptor. Collectively, these mechanisms provide a biologically plausible explanation for the transition from localized venous thrombosis to diffuse systemic thrombo-inflammatory disease observed in the present patient. Impairment of this pathway may further amplify endothelial injury, vascular permeability, leukocyte adhesion, and thrombo-inflammatory responses, thereby contributing to the progression from localized thrombosis to systemic coagulopathy and organ dysfunction [32,33,34].

Our patient demonstrated markedly reduced Protein C activity (28.6%) during acute consumptive illness, together with markedly elevated vWF activity and antigen (>500%). In the absence of convalescent repeat testing, this finding does not establish hereditary Protein C deficiency. These findings are consistent with marked endothelial activation occurring in the setting of severe thrombo-inflammatory disease but are not specific for endothelial dysfunction, as both abnormalities may also reflect consumptive coagulopathy and an acute-phase response. Because ADAMTS13 activity was not measured, no specific alteration of the vWF–ADAMTS13 axis can be inferred. Importantly, Protein S and antithrombin activities remained within the reference range, suggesting that impairment of the Protein C pathway represented the predominant endogenous anticoagulant abnormality rather than generalized depletion of all physiological anticoagulant systems [12].

These findings provide a biologically plausible explanation for the thrombo-inflammatory phenotype but do not establish a causal relationship with the preceding influenza-like illness.

### 4.3. Thrombotic-Predominant Overt Disseminated Intravascular Coagulation

Overt disseminated intravascular coagulation (DIC) is increasingly recognized as a heterogeneous thrombo-inflammatory syndrome characterized by dysregulated interactions between inflammation, endothelial activation, and coagulation. Depending on the underlying trigger, distinct clinical phenotypes may develop, ranging from predominantly hemorrhagic to predominantly thrombotic manifestations [5,8,35].

Importantly, extensive pulmonary embolism and iliofemoral thrombosis were documented on 19 January, whereas overt DIC developed on 2–3 February. Overt DIC therefore cannot be considered the established cause of the initial thrombosis. Progressive thrombotic burden, severe venous ischemia, compartment syndrome, fasciotomy, tissue injury, and shock may have promoted the evolution toward consumptive coagulopathy. The subsequently suspected secondary infection may have further amplified this process. Accordingly, overt DIC was interpreted as a later amplifier of an already established thromboinflammatory syndrome.

The pathophysiological hallmark of this process is the simultaneous presence of ongoing thrombosis and consumption of coagulation factors. Progressive thrombocytopenia, markedly elevated D-dimer concentrations, prolonged coagulation times, hypofibrinogenemia, and reduced activity of physiological anticoagulants have consistently been associated with disease severity and adverse outcomes in patients with overt DIC and critical illness [36,37,38,39]. The laboratory profile observed in our patient closely reflects this pathophysiological model.

The serial evolution in our patient supports dynamic progression to overt DIC rather than the presence of DIC throughout hospitalization, consistent with the updated ISTH framework [11]. Serial investigations demonstrated progressive thrombocytopenia, persistently elevated D-dimer levels, severe hypofibrinogenemia, prolonged PT/INR, markedly reduced Protein C activity, and decreased factors II, VII, and XIII activities. According to the International Society on Thrombosis and Haemostasis (ISTH) overt DIC scoring system, the patient fulfilled the diagnostic criteria for overt disseminated intravascular coagulation, with a calculated score of 7 based on severe thrombocytopenia (41 × 10^9^/L), markedly elevated D-dimer levels, prolonged prothrombin time, and profound hypofibrinogenemia (62.2 mg/dL) [11]. When interpreted together with extensive multisite thrombosis, circulatory shock requiring vasopressor support, and multiple organ dysfunction, these findings are most consistent with a thrombotic-predominant phenotype of overt disseminated intravascular coagulation.

Importantly, the coexistence of extensive thrombosis and laboratory evidence of consumptive coagulopathy highlights that thrombosis and consumption are not mutually exclusive phenomena but complementary manifestations of the same dysregulated thrombo-inflammatory process. This concept is increasingly emphasized in contemporary models of overt DIC, which recognize thrombotic and hemorrhagic phenotypes as part of a dynamic disease spectrum. Overall, the clinical and laboratory findings observed in our patient closely align with this thrombotic-predominant phenotype of overt DIC [40,41].

### 4.4. Positive Anti-PF4/Heparin Antibodies: Clinically Significant HIT or an Epiphenomenon?

The interpretation of positive anti-PF4/heparin antibodies represented one of the major diagnostic challenges in this case. Current guidelines emphasize that HIT cannot be diagnosed on the basis of a positive immunoassay alone, particularly in critically ill patients with sepsis, major surgery, or disseminated intravascular coagulation (DIC), where false-positive results and overlapping clinical features are well recognized [42,43,44]. Consequently, distinguishing clinically significant HIT from non-platelet-activating anti-PF4/heparin antibody positivity in a patient with evolving thromboinflammatory coagulopathy may be particularly challenging in critically ill patients [1].

In the present case, serum for anti-PF4/heparin testing was collected on 26 January 2026, at 12:30, after seven days of LMWH exposure and before continuous intravenous UFH was initiated at 21:00. The external reference laboratory reported a qualitative positive result on 5 February 2026. However, the available report did not specify the assay manufacturer, analytical platform, immunoglobulin specificity, optical density, or positivity threshold, and no functional platelet-activation assay was performed. Thus, the result raised a clinically relevant suspicion of HIT but did not establish the presence of platelet-activating antibodies or confirm the diagnosis.

Anti-PF4 antibodies may occur in inflammatory and thrombotic conditions outside classical HIT, and immunoassay positivity does not necessarily demonstrate platelet-activating antibodies [45,46]. Therefore, positive anti-PF4/heparin immunoassays should be interpreted together with the timing of heparin exposure, platelet-count kinetics, thrombotic evolution, competing causes of thrombocytopenia, and functional platelet-activation testing when available [22,47,48].

In our patient, the platelet count decreased from a post-admission peak of 213 × 10^3^/µL on 20 January to 131 × 10^3^/µL on 26 January, when the anti-PF4 sample was collected following seven days of LMWH exposure. Progressive thrombosis and qualitative positive immunoassay raised a clinically relevant suspicion of HIT. However, thrombocytopenia had been present before heparin exposure, while severe inflammation, extensive thrombosis, tissue ischemia, fasciotomy, and evolving consumptive coagulopathy represented important competing explanations. The 4Ts score at the time of sampling was 4, indicating intermediate pretest probability. The subsequent development of severe hypofibrinogenemia, markedly elevated D-dimer levels, coagulation-factor consumption, and overt DIC supported a predominant consumptive coagulopathy but did not exclude concomitant HIT [49,50]. Without functional platelet-activation testing, HIT could neither be confirmed nor definitively excluded. The case-specific findings are summarized in Table 3.

### 4.5. Additional Differential Diagnostic Considerations

The extensive left iliofemoral thrombosis, massive edema, progressive ischemia, compartment syndrome, and initially preserved arterial axis were retrospectively compatible with phlegmasia cerulea dolens progressing to venous gangrene [51,52]. Although not formally diagnosed at presentation, this mechanism plausibly explains the severe limb injury and subsequent amputation.

May–Thurner syndrome was considered because of the left iliofemoral thrombosis in a young woman [53,54]. However, iliac-vein compression was not reported on the available CT examinations, and neither dedicated venous imaging nor intravascular ultrasound was performed. It could therefore neither be confirmed nor excluded but would not explain the widespread thrombotic involvement.

The central venous catheter was placed on 25 January, before the later superior vena cava, right atrial, and right internal jugular thromboses. A catheter-associated contribution to these events is possible [55,56]. However, catheter exposure cannot explain the iliofemoral thrombosis and pulmonary embolism documented on 19 January.

Thrombotic microangiopathy/TTP was also considered. The blood smear on 21 January showed only rare schistocytes within a predominantly microcytic, hypochromic pattern. LDH peaked at 1538 U/L concurrently with CK exceeding 12,000 U/L and extensive ischemic tissue injury, limiting its specificity for hemolysis. Bilirubin remained normal and renal function was preserved; haptoglobin and ADAMTS13 activity were unavailable. Although TTP could not be definitively excluded without ADAMTS13 testing [57,58], the absence of documented microangiopathic hemolytic anemia and the presence of prolonged PT, severe hypofibrinogenemia, markedly elevated D-dimer, and coagulation-factor consumption favored overt DIC.

### 4.6. Integrative Pathophysiological and Clinical Implications

This case illustrates the interaction between extensive thrombosis, endothelial activation, tissue ischemia, and consumptive coagulopathy in a critically ill patient [59,60,61].

Anti-PF4 antibody positivity may occur outside classical HIT, and a positive immunoassay alone does not establish clinically significant, platelet-activating HIT [22,48,62,63]. In this case, the subsequent development of severe consumptive coagulopathy and fulfillment of the ISTH criteria supported overt DIC as the predominant coagulation disorder, while concomitant HIT could not be definitively excluded.

Management required balancing ongoing thrombosis, bleeding risk, and the need for urgent surgical intervention [64,65,66]. UFH was continued because it permitted rapid dose adjustment in the perioperative setting and because the overall evolution was considered more consistent with overt DIC than isolated HIT. Platelet-count recovery despite continued UFH weighed against persistent platelet-activating HIT but did not definitively exclude it in the absence of functional testing.

More broadly, severe systemic inflammation, extensive tissue injury, possible infection, and endothelial activation may simultaneously promote immunothrombosis and consumption of platelets and coagulation factors. Recognition of this dynamic evolution and integration of clinical, laboratory, imaging, and temporal data are essential for the individualized management of complex thromboinflammatory syndromes [67,68].

## 5. Limitations

Several limitations should be acknowledged. The presumed influenza-like illness could not be microbiologically confirmed, as molecular testing was performed late after symptom onset and yielded a negative result. In addition, functional confirmatory assays for HIT (SRA/HIPA) and biomarkers such as NET formation markers, complement activation, and ADAMTS13 activity were not available. Furthermore, the extended coagulation assessment was performed six and a half hours after the final enoxaparin dose and before UFH initiation. Substantial residual heparin activity was nevertheless present, as indicated by anti-Xa activity > 1.50 IU/mL and thrombin time > 160 s, limiting the interpretation of heparin-sensitive clot-based assays, particularly APCR and factor XI activity. Therefore, the proposed mechanistic interpretation should be regarded as biologically plausible rather than definitively established.

## 6. Conclusions

In conclusion, this report describes a fulminant thromboinflammatory syndrome following an influenza-like illness of unconfirmed etiology, characterized by extensive multisite thrombosis, laboratory evidence of endothelial activation, and the subsequent development of overt DIC with a thrombotic-predominant phenotype. The focused literature review contextualizes the interplay between immunothrombosis, endothelial activation, and dysregulated coagulation, as well as the diagnostic overlap between overt DIC and anti-PF4/heparin antibody positivity. Although HIT remained clinically plausible, the qualitative immunoassay result without functional platelet-activation testing did not confirm the diagnosis. This case underscores the importance of serially integrating clinical evolution, platelet-count kinetics, coagulation parameters, heparin exposure, and imaging findings when evaluating complex thromboinflammatory conditions.

## Figures and Tables

**Table 1:** Serial laboratory parameters throughout hospitalization.

Parameter	Infectious Diseases (16–18 Jan)	Cardiology (20–24 Jan)	ICU I/Pre-Amputation (26 Jan–13 Feb)	ICU II/Post-Amputation (16–23 Feb)	Recovery/Discharge (24 Feb–10 Mar)
WBC, ×10^9^/L	9.71–11.07	10.36–24.33	3.73–32.77	8.87–11.15	5.56–14.94
Hemoglobin, g/dL	8.3–8.4	7.3–7.9	6.9–8.7	8.8–9.7	8.3–11.5
Platelets, ×10^9^/L	89–163	136–213	41–262	200–305	186–359
PT, s	14.9–16.4	14.4–16.7	14.3–19.4	15.9–19.0	18.9
INR	1.34–1.39	1.30–1.41	1.29–1.75	1.34–1.72	1.61
aPTT, s *	—	29.3	27.7–107.9	32.3–96.1	54.0
Fibrinogen, mg/dL	122.0–342.9	175.6–425.4	62.2–371.2	407.9–474.8	340.0–582.0
D-dimer, mg/L	Positive (no numeric value)	—	16.20–35.20	4.52	6.40
CRP, mg/L	161.7–396.1	271.0–371.9	118.1–281.1	66.5–202.3	30.3–203.4
Procalcitonin, ng/mL	—	—	>10	—	<0.5
AST, U/L	12–14	—	24–366	15–41	26–41
ALT, U/L	13–15	—	25–214	12–24	23–33
CK, U/L	34	36	448–12,227	—	17–31
Albumin, g/L	—	—	18.1	28.8	31.4–40.2
Creatinine, mg/dL	0.51–0.53	0.45–0.52	0.39–0.57	0.38–0.48	0.37–0.55

Notes: Values are presented as the observed ranges within each major clinical phase of hospitalization. * aPTT values should be interpreted in the context of continuous intravenous unfractionated heparin therapy. Peak or nadir values are included within each hospitalization phase to illustrate the dynamic evolution of disease severity. Numeric D-dimer values were unavailable during the initial hospitalization because the assay was reported qualitatively. Abbreviations: ICU, intensive care unit; PT, prothrombin time; INR, international normalized ratio; aPTT, activated partial thromboplastin time; CRP, C-reactive protein; PCT, procalcitonin.

**Table 2:** Extended coagulation and thrombophilia assessment.

Parameter	Patient Result	Reference Range
**Global coagulation**		
Prothrombin time (PT)	20.3 s	11.5 s
INR	1.81	0.8–1.2
Thrombin time	>160.0 s	16.0–18.3 s
D-dimer	24.46 mg/L	<0.50 mg/L
Anti-factor Xa activity	>1.50 IU/mL	0.60–1.00 IU/mL
**Natural Anticoagulant Pathways**		
Protein C activity	28.6%	70.0–131.0%
Protein S activity	96.3%	62.0–126.0%
Free Protein S (antigen)	78.4%	64.7–115.3%
Antithrombin activity	96.8%	90.0–119.0%
Activated Protein C Resistance (APCR)	1.55	≥2.10
**Coagulation Factors**		
Von Willebrand factor		
vWF activity	513.1%	49.5–187.0%
vWF antigen	500.7%	56.0–160.0%
Factor VII activity	46.2%	75.0–130.0%
Factor VIII activity	83.9%	80.0–166.0%
Factor IX activity	112.6%	60.0–117.0%
Factor X activity	130.1%	73.0–128.0%
Factor XI activity	<0.7%	72.0–122.0%
Factor XII activity	52.9%	44.0–116.0%
Factor XIII activity	52.0%	64.0–133.0%
**Antiphospholipid Panel**		
Lupus anticoagulant	Absent	Absent
Antiphospholipid IgG	Negative	Negative (<10.0 GPL-U/mL)
Antiphospholipid IgM	Negative (2.7 MPL-U/mL)	Negative (<10.0 MPL-U/mL)
Anticardiolipin IgG	Negative (4.2 GPL-U/mL)	Negative (<10.0 GPL-U/mL)
Anticardiolipin IgM	Negative (2.4 MPL-U/mL)	Negative (<7.0 MPL-U/mL)
Anti-β2 glycoprotein I IgG	Negative (3.2 U/mL)	Negative (<5.0 U/mL)
Anti-β2 glycoprotein I IgM	Negative (2.80 U/mL)	Negative (<5.0 U/mL)
**Anti-Pf4/Heparin Immunoassay**		
PF4/heparin antibodies	Positive, qualitative immunoassay (sample collected 26 January; result reported 5 February 2026)	Negative
**Genetic Thrombophilia**		
Factor V Leiden (G1691A)	Absent	-
Factor V H1299R (R2)	Absent	-
Factor II G20210A	Absent	-
Factor XIII V34L	Absent	-
Homocysteine	7.13 μmol/L	4.92–11.88 μmol/L

Notes: Samples were collected on 26 January at 12:30, after the final enoxaparin dose at 06:00 and before UFH initiation at 21:00. The marked heparin effect at sampling (anti-Xa > 1.50 IU/mL; thrombin time > 160 s) limits the interpretation of susceptible clot-based assays. APCR was considered unreliable because of heparin interference. Protein C activity of 28.6% was interpreted as markedly reduced during acute illness; without convalescent repeat testing, hereditary deficiency cannot be established. The qualitative anti-PF4/heparin result was reported on 5 February; no functional platelet-activation assay was performed. Abbreviations: APCR, activated protein C resistance; INR, international normalized ratio; PF4, platelet factor 4; PT—prothrombin time; UFH—unfractionated heparin; vWF—von Willebrand factor.

**Table 3:** Structured comparison of overt disseminated intravascular coagulation and heparin-induced thrombocytopenia in relation to the present case.

Diagnostic Feature	Present Case	Overt DIC	HIT
Temporal evolution	Initial DVT and PE on 19 January; overt DIC on 2–3 February	Dynamic progression following systemic inflammation, tissue injury or shock	Typically begins 5–10 days after heparin exposure
Platelet kinetics	213 to 131 × 10^9^/L at anti-PF4 sampling, a 38.5% decrease; subsequent nadir 41 × 10^9^/L	Progressive thrombocytopenia caused by consumption	Typically >50% decrease; nadir usually ≥20 × 10^9^/L
Thrombotic manifestations	Initial DVT and PE preceded anticoagulation; subsequent multisite progression; right internal jugular thrombosis after central venous catheter placement	Microvascular and/or macrovascular thrombosis may occur	New or progressive thrombosis after heparin exposure
PT	17.1 s	Frequently prolonged	Usually normal in isolated HIT
Thrombocytopenia before heparin exposure	Thrombocytopenia was already present before heparin exposure. Platelet count was 89–163 × 10^9^/L before the first enoxaparin dose.	Compatible with pre-existing inflammatory or consumptive thrombocytopenia.	Weighs against HIT as the initial cause of thrombocytopenia.
Fibrinogen	62.2–62.7 mg/dL	Reduced in overt consumptive coagulopathy	Usually preserved in isolated HIT
D-dimer	35.20 mg/L	Markedly elevated	May be elevated in association with thrombosis
Coagulation factors and Protein C	Reduced factors II, VII and XIII; Protein C activity 28.6% during acute illness	Compatible with consumption and reduced endogenous anticoagulant activity	Not characteristic of isolated HIT
ISTH overt DIC score	7 on 2–3 February	Fulfills laboratory criteria for overt DIC	Not applicable
Anti-PF4/heparin immunoassay	Qualitative positive result; sample collected after LMWH and before UFH	Not included in DIC diagnostic criteria	Compatible with HIT but not diagnostic in isolation
Functional platelet-activation assay	Not performed	Not applicable	Required for laboratory demonstration of platelet-activating antibodies
4Ts score	Thrombocytopenia, 1; timing, 2; thrombotic progression, 1; alternative causes, 0; total, 4	Not applicable	Intermediate pretest probability
Competing causes of thrombocytopenia	Severe inflammation, tissue ischemia, surgery, shock and subsequent overt DIC	Recognized causes or consequences of consumptive coagulopathy	Reduce clinical specificity for HIT but do not exclude it

Abbreviations: DIC, disseminated intravascular coagulation; DVT, deep-vein thrombosis; HIT, heparin-induced thrombocytopenia; ISTH, International Society on Thrombosis and Haemostasis; LMWH, low-molecular-weight heparin; PE, pulmonary embolism; PF4, platelet factor 4; PT, prothrombin time; UFH, unfractionated heparin. Notes: Comparative characteristics are based on established ISTH DIC and HIT diagnostic frameworks. The table is intended for structured comparison and does not replace validated diagnostic algorithms.

## Data Availability

The data supporting the findings of this case report are available from the corresponding author upon reasonable request.

## Supplementary Materials

The following supporting information can be downloaded at: https://www.mdpi.com/article/10.3390/ijms27167456/s1.

## Abbreviations

The following abbreviations are used in this manuscript:

DIC	disseminated intravascular coagulation
HIPA	heparin-induced platelet activation assay
HIT	heparin-induced thrombocytopenia
ICU	intensive care unit
ISTH	International Society on Thrombosis and Haemostasis
NETs	neutrophil extracellular traps
OD	optical density
PF4	platelet factor 4
SIC	sepsis-induced coagulopathy
SRA	serotonin release assay
SSC	Scientific and Standardization Committee
UFH	unfractionated heparin
vWF	von Willebrand factor
