# Supplementary material for: Fulminant Thromboinflammatory Syndrome Following an Influenza-like Illness in an Adolescent: Clinical Insights from a Case Report
Source: Int J Mol Sci. 2026 Aug 20;27(16):7456. doi: 10.3390/ijms27167456 (PMC13513553; doi:10.3390/ijms27167456)
Supplement: Supplementary file 1 [file ijms-27-07456-s001.zip › ijms-4480703-supplementary.pdf]

Table S1. Chronological timeline of major clinical events and therapeutic interventions

Date	Major clinical event	Objective findings	Therapeutic decision
**~10 Jan 2026**	Onset of influenza-like illness	Fever, myalgia, respiratory symptoms	Symptomatic treatment
**15 Jan 2026**	Hospital admission after influenza-like illness	Persistent fever and dyspnea	Diagnostic evaluation
**19 Jan 2026**	Extensive venous thromboembolism confirmed	Left iliofemoral DVT and pulmonary embolism	Therapeutic enoxaparin initiated
**25 Jan 2026**	Acute limb ischemia	Extensive venous thrombosis with compartment syndrome	Emergency vascular surgery (fasciotomy)
**26 Jan 2026**	Admission to ICU	Post-fasciotomy, compartment syndrome, bilateral PE, critical limb ischemia	Intensive care management
**27 Jan 2026**	Anticoagulation reassessment	Subtherapeutic aPTT	UFH bolus and dose escalation
**28 Jan 2026**	Follow-up vascular evaluation	Partial venous recanalization despite persistent thrombotic disease	Continued anticoagulation
**4 Feb 2026**	Repeat Angio-CT	Progressive thoracic and abdominal thrombosis	Continued multidisciplinary management
**13 Feb 2026**	Vascular surgery consultation	Gangrene of the foot and distal leg	Indication for major amputation
**13 Feb 2026**	Transfer	Transfer to Vascular Surgery	Surgical management
**16 Feb 2026**	Postoperative ICU admission	Status after major lower limb amputation	Intensive care
**19 Feb 2026**	Repeat Angio-CT	Persistent extensive thrombosis involving cervical veins	Continued evaluation
**23 Feb 2026**	Transfer	Clinical stabilization	Transfer to Cardiology
